# The management of complex periprosthetic humeral fractures: a case series of strut allograft augmentation, and a review of the literature

**DOI:** 10.1007/s11751-013-0155-x

**Published:** 2013-02-28

**Authors:** Alex J. Trompeter, Rohit R. Gupta

**Affiliations:** Rowley Bristow Department of Trauma and Orthopaedics, St. Peter’s Hospital, Guildford Road, Chertsey, Surrey, KT16 0PZ UK

**Keywords:** Periprosthetic, Fracture, Humerus, Allograft, Strut, Reverse geometry

## Abstract

There is little published discussion on the management of postoperative periprosthetic humeral fractures where rotator cuff function is poor, the bone stock is dwindling or both. This is a phenomenon increasingly seen in the older, more osteoporotic population and presents an interesting challenge especially in when faced with these patients with poor bone quality. We present the treatment of three fractures with the use of long-stem reverse geometry arthroplasty and other surgical techniques more commonly reserved for periprosthetic fractures of the proximal femur such as cortical strut allograft augmentation. We believe revision to reverse geometry long-stem implant with cortical strut allograft augmentation to be safe and appropriate in the management of these complex injuries, although technically challenging, and has excellent initial and medium-term results.

## Introduction

Much is written about the management of intra-operative periprosthetic humeral fractures. Postoperative fractures where the stemmed humeral implant is stable and shoulder function is good are described, although less frequently. However, very little is discussed on the management of humeral fractures where rotator cuff function is poor, the bone stock is dwindling or both. This phenomenon is increasingly seen in the older, more osteoporotic population and presents an interesting challenge especially in when faced with these patients with poor bone quality. Osteoporotic fractures (periprosthetic or not) are likely to remain one of the largest challenges in orthopaedics for the next few years. Techniques of fixation and management are different to those fractures in good quality bone and thus require improved understanding and a drive for innovation in their management.

Periprosthetic humeral fractures have an incidence of between 0.6 and 3 % in all shoulder arthroplasty [1, 2]. They account for approximately 11 % of all complications related to total shoulder arthroplasty [3]. The treatment options include conservative (non-operative) and surgical management, depending on fracture and patient personality. There is a significant patient morbidity associated with these injuries, and periprosthetic fractures are less likely to unite than those humeral fractures not associated with arthroplasty [4].

This article aims to review the current literature on the management of these complex cases. As well as a review of the literature surrounding the management of periprosthetic humeral fractures, we present a case series of three fractures with the use of long-stem primary or revision arthroplasty and a surgical technique more commonly reserved for periprosthetic fractures of the proximal femur—cortical strut allograft augmentation.

## Review of the literature

### Shoulder arthroplasty

The glenohumeral joint is a poorly constrained ball- and socket-type joint. It is subject to the processes of degenerative change (osteoarthritis), trauma and inflammatory arthropathy in the same manner as other large synovial joints of the body. Treatment for these conditions ranges from conservative to surgical and includes hemiarthroplasty and total arthroplasty.

The principal of shoulder arthroplasty was first described as long ago as 1893, with Pean’s constrained total shoulder arthroplasty. Neer popularised the hemiarthroplasty in 1951, and several generations have passed to where we are today [5]. The principals have remained the same although the development of reverse geometry implants in the latter part of the twentieth century has offered a significant advance in the management of patients with rotator cuff insufficiency requiring arthroplasty.

For as long as shoulder replacement has been in use, the complication of periprosthetic fracture has been present. As our population ages, patient demands increase, implants develop and surgical skill improves, the use of these prostheses will expand. As such, the prevalence of fractures surrounding these devices is likely to increase. The older and higher demand population bring with them unique problems in terms of poorer bone quality and a higher incidence of rotator cuff arthropathy.

### Intra-operative humeral fractures

These fractures of the humerus (usually the shaft) are a well-recognised risk of shoulder arthroplasty. These fractures account for approximately three quarters of all periprosthetic humeral fractures. Campbell et al. [6] showed that half of these occurred in the diaphyseal region of the humeral shaft and classified these intra-operative fractures according to anatomical region. Their study highlighted that many fractures could be attributed to poor surgical techniques, referring to excessive torque on the humerus from reaming or external rotation manoeuvres intra-operatively [1, 7]. Fractures occur more frequently in total shoulder arthroplasty than hemiarthroplasty, due to the need to access the glenoid and thus increase rotation on the humerus [8]. Generally, it is accepted that the management of these sorts of fractures is best met with longer stem prostheses and cerclage wiring if required [5].

### Postoperative periprosthetic humeral fractures

Generally, these are much rarer than intra-operative fractures. A review by Worland in 1999 showed that only 30 reported cases of postoperative fractures could be found in the literature, with little consensus on how to manage them [8]. By 2008, only 51 cases were reported, in 9 articles, and there was still a poor body of knowledge and evidence on how to best manage the more complex fractures [7].

The fractures can be classified based upon fracture anatomy and implant stability, much in the same way the Vancouver system is used in the classification of periprosthetic proximal femoral fractures. The classification differs slightly from that of the intra-operative periprosthetic fractures. Type A postoperative periprosthetic humeral fractures occur around the tuberosities. Type B fractures occur around the stem. These can be subclassified into type B1—spiral fractures with a stable implant; B2—short oblique fractures at the tip of the stem with a stable implant; B3—fractures about the stem with an unstable implant. Type C fractures occur well distal to the tip of the stem [8]. This system, described in 1999 by Worland, is a modification of the University of Texas at San Antonio classification [1].

### Simple fractures

**Fig. 1:**
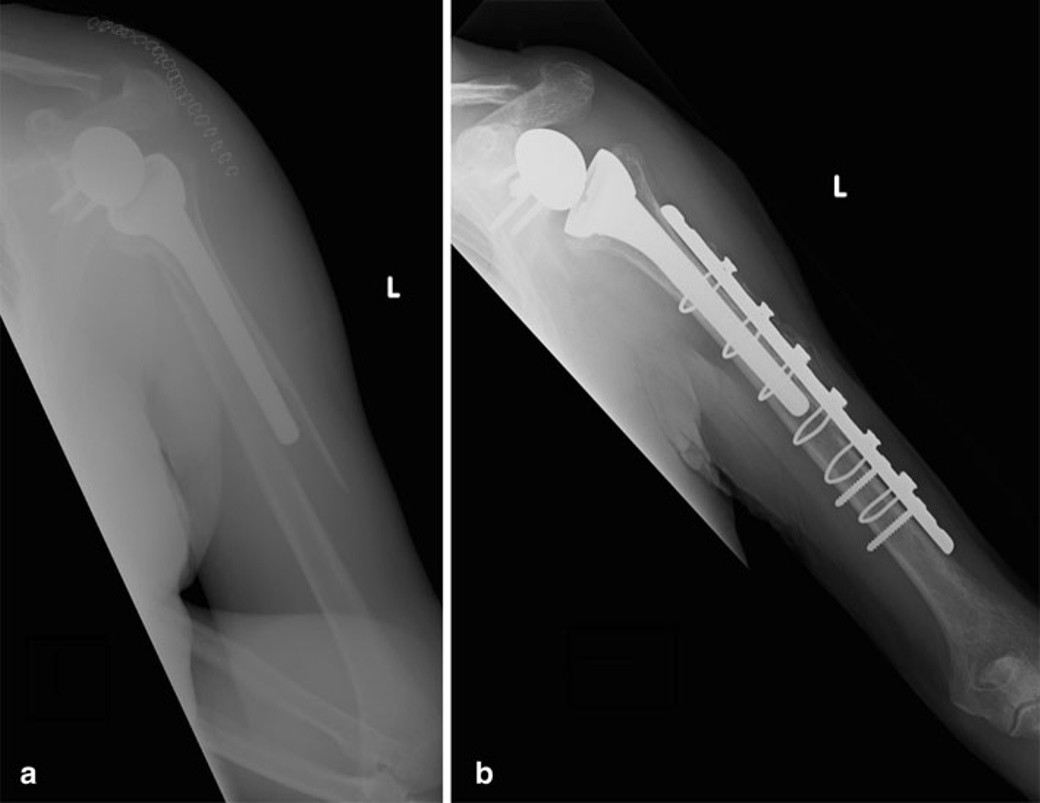
A type B1 postoperative periprosthetic fracture (**a**) in a patient who fell 1 week after surgery, treated with cable plating (**b**)

Type A and B1 fractures can be considered as simple injuries, as can those fractures related to shoulder resurfacings. Careful management is still required with adequate attention to both patient and fracture personality. However, they are less often complicated by poor bone quality. Spiral fractures around well-fixed prostheses are typically managed with cerclage wiring or a cable plate system (Fig. 1a, b). These may or may not require revision to longer stem implants. Good results have been achieved with this method as described by Campbell et al. Fractures of the proximal humeral metaphysis can be treated with standard stem arthroplasty and cerclage wiring if the stem extends distal to the fracture site by at least 3 cortical diameters. Campbell showed that anatomic reduction of fractures treated by surgical means results in shorter healing times [6]. Long oblique and spiral fractures can be successfully treated non-operatively, provided that the skeletal alignment is acceptable [9]. Further distally, fractures are often treated conservatively or managed as for other distal humeral fractures [2].

### Complex fractures

What do we mean by complex fractures? This should be a term reserved for those periprosthetic fractures that are usually within the diaphysis of the humerus either at the level of or just beyond the tip of the implant stem. With reference to fracture pattern, transverse and short oblique fractures are typically more complicated to treat in that they offer less biomechanical stability and a smaller fracture surface area over which to achieve union. Spiral fractures tend to allow for simpler fixation techniques. Conservative, non-operative measures have no real role in the management of these injuries: all reported cases treated non-operatively have progressed to delayed or non-union and required operative intervention [7].

Complex periprosthetic fractures typically involve or are compounded by issues such as the absence of a functioning rotator cuff, osteoporotic bone or limited proximal bone stock, and loose implants and usually require revision surgery. Very little is known about the best way to manage these rarer periprosthetic fractures. And as is so often the way in medicine, we can be presented with the worst-case scenarios, that is, patients with more than one of the above complex features. This is the area which needs researching. As patients (and their implants) live longer, and as implants are developed and shoulder arthroplasty grows in popularity, these complex fractures will continue to arise and require advances in their management.

Type B2 and B3 fractures have typically been referred to as the more complex or difficult to treat. It is commonly accepted that management of the fracture is best achieved with a revision to longer stem prostheses. Fractures at the level of the implant are typically treated with revision arthroplasty if the implant is loose or if the fracture overlaps a significant portion of the implant. It is recommended that well-fixed components with periprosthetic fractures at the tip of the prosthesis are treated with internal fixation [10, 11] including with cable plate systems [12].

Wright showed that B2 fractures are extremely slow to unite if treated conservatively and in fact may never heal [9]. Long-stem intramedullary fixation with revision implant and cerclage wiring has been the preferred surgical option for treatment of unstable humeral shaft fractures up to now [6]. It is also suggested that fractures resulting in prosthetic instability should be treated with a long-stem prosthesis extending at least 2–3 cortical diameters past the fracture site with consideration for rigid plate fixation [1, 7]. Diaphyseal fractures that were treated with standard stem arthroplasty with or without supplemental fixation had a longer time to fracture union, a higher complication rate and prolonged rehabilitation [6]. Similar management of similar injuries by Kumar et al. [10] highlighted an average time to union of 278 days, and this study did not advocate revision to longer stem components unless already loose.

Comminuted and open injuries offer unique challenges. Open fractures and those associated with nerve injury typically require surgical intervention, followed by management appropriate for that injury. Comminuted fractures are rare, possibly due to the osteopenic bone seen in the patient group who have humeral implants [9].

### Management for those with an absence of a functional cuff

A functioning rotator cuff is required for normal glenohumeral motion. Absence of a cuff leads to superior escape of the humeral head, eccentric positioning of the humerus within the glenoid and poor glenohumeral control. This leads to erosion of the glenoid and proximal humeral bone loss, along with subacromial sclerosis. Revision of failed hemiarthroplasty due to rotator cuff arthropathy/deficiency to reverse geometry shoulder prosthesis is a well-recognised management option and has good short-term results [13, 14]. There is little long-term follow-up of reverse geometry implants both as a primary and revision procedure for cuff-deficient shoulders, although early to mid-term outcomes are encouraging [15].

There is very little literature on the management of periprosthetic humeral fractures in the absence of a functioning rotator cuff. The management of patients with pre-existing rotator cuff insufficiency who suffer fractures around cemented proximal humeral implants remains relatively controversial. McDonough and Crosby make the very valid point that treatment decisions should be made with respect to obtaining fracture stability, initiating early glenohumeral motion and restoring shoulder function [1]. A cuff-deficient shoulder will not allow this. In the elective orthopaedic setting, revision arthroplasty is often to reverse polarity total shoulder implants if the patient has signs of rotator cuff deficiency [15].

### Patient factors

It is of course imperative to remember the patient factors that can influence management decisions and fracture healing. Nutritional status, systemic disease (rheumatoid arthritis, diabetes mellitus, cardiac and respiratory disease), steroid medications, smoking and mental status are all important factors. A nursing home resident who is already fully dependent on carers will require a different management approach to that of an independent patient still in employment. Poor bone quality, advancing age, female sex and rheumatoid arthritis are the principal predictors for periprosthetic fracture [1, 2].

In the series of patients treated by Campbell, mild osteopenia was present in 45 % of the patients, whereas 30 % had severe osteopenia [6]. This has an important influence on fracture healing, as well as the quality and suitability of bone for surgical fixation. Locking plate technology has allowed for fixation of osteopenic bone but the use of unicortical screws in a plating system for the management of a B2-type fracture would unlikely provide an adequately stable construct.

Obese patients and female patients with large breasts are also at risk of displacing fractures if being managed conservatively. Large breasts tend to abduct the proximal humerus and produce a varus deformity at the fracture sites, and obese arms are notoriously difficult to apply functional braces.

### Biological strut graft

Is revision to longer stem prosthesis in type B2 and B3 fractures enough? We ask whether this provides an appropriate strain environment for fracture union. Is relying simply on distal fit a suitable option? Is cerclage wiring directly onto the humeral surface appropriate?

In asking these questions, we looked at the use of strut allograft augmentation in the management of periprosthetic femoral fractures and transposed this technique to humeral fractures. It is agreed as above that revision to long-stem implants is required in unstable fracture configurations. Whether these are normal or reverse geometry implants remains to be decided. However, we feel that relying solely on the distal hold of a revision prosthesis to support the fracture is inappropriate. Cable plate systems do offer further support, but the use of a biological construct is more appealing. This is discussed in detail below. The use of cortical onlay allografts or biological strut grafts is well described in the management of periprosthetic fractures around the hip, and we feel that using this approach to periprosthetic fracture management in the humerus is appropriate.

The report by Kumar et al. [10] did show the use of cortical strut allograft in the management of a type B1 fracture; however, no revision of the humeral component was performed. Their fracture united at 4 months. This was a long spiral fracture and likely to unite with internal fixation, and the role of strut allograft in their case therefore is questionable.

We can find no reported descriptions of revision to long-stem reverse geometry implants and augmentation with cortical strut allograft in fracture management. Levy et al. [13] do describe the use of cortical allograft in restoring the deltoid contour and working length when revising failed hemiarthroplasty to reverse geometry implants, but this is in the elective orthopaedic setting. Vascularised fibular graft has also been described in the management of non-unions of periprosthetic humeral fractures [9]. This did not provide mechanical support but rather served to improve the biological environment for a fracture that was struggling to unite.

Sanchez-Sotello described the management of 11 periprosthetic humeral fractures around elbow arthroplasty with revision to the Coonrad-Morrey semiconstrained elbow arthroplasty and augmentation with cortical strut allograft. They showed excellent union results and good functional outcome although their complication rate was substantial [16]. They typically used two struts in the management of their fractures and make the valid points that the struts must be of sufficient length to span the fracture site and allow for good proximal and distal hold.

We have had good results in cases treated with reverse geometry long-stem implants in the management of complex primary and periprosthetic fractures. Furthermore, we describe a new previously unreported approach to this problem of cuff insufficiency in the presence of a (periprosthetic) humeral fracture by incorporation of a biological strut graft to our reverse geometry revision construct.

Below are a description of our operative technique and a summary of the cases treated up until now.

## Cortical strut allograft—case series

### Operative technique

**Fig. 2:**
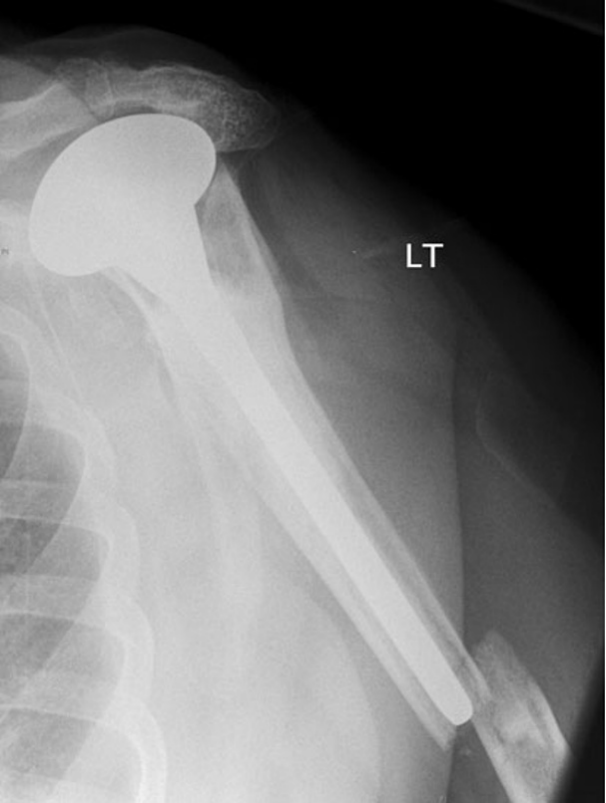
Plain AP radiograph showing Type B2 periprosthetic fracture of left humerus (case 1), with cemented Neer hemiarthroplasty in situ. Note the superior migration of the humeral head and subacromial sclerosis implying pre-existing rotator cuff deficiency as well as the osteoporotic bone of the humerus

We detail the preferred operative technique of the senior author (RRG). Two cases of periprosthetic fracture and one additional humeral fracture below a previous malunion of a proximal humerus fracture were all treated with reverse geometry long-stem implant and augmentation with biological cortical strut allograft and cables. All patients were assessed with history and examination in the outpatient department. All patients had been referred for specialist opinion after acute admission at the time of their fracture and subsequent failed non-operative management of the patient. Background medical history as well as pre-existing functional level was taken into account. All patients had several diagnostic imaging studies to confirm rotator cuff insufficiency and aid surgical planning. Plain radiographs were used to classify the fracture, assess the general bone quality and the severity of cuff arthropathy with superior migration of the prosthesis (Fig. 2) or humeral head. Computed tomography (CT) and ultrasound scan (USS) were used to assess the glenoid bone stock and confirm the absence of a functional rotator cuff in all cases thus signalling the need for reverse geometry arthroplasty in the treatment plan.

**Fig. 3:**
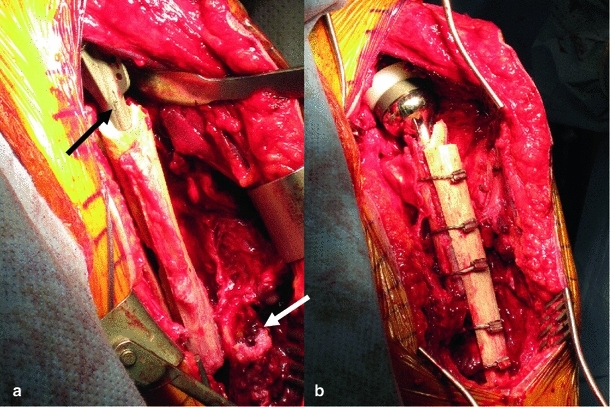
Intra-operative photographs from case 1 showing (**a**) extended deltopectoral approach, well-fixed cemented implant (*black arrow*) and fracture at the level of the tip of the prosthesis (*white arrow*) and (**b**) demonstrating the reverse geometry implant in situ, with cortical strut allograft support fixed with cable tie system

After discussion with the patients and relatives, decisions were made to manage the fractures surgically. All patients were optimised for theatre, and fully informed consent was gained. Theatre set up was as for the lead surgeon’s (RRG) preference for shoulder surgery with the patient in a beach chair position. An extended deltopectoral approach was used, and the proximal humerus, implant and fracture were all exposed (Fig. 3a). Neurovascular structures were identified and protected throughout. The extent rotator cuff deficiency was confirmed at this stage. In the case of the periprosthetic fractures, the implants were removed by an extended humeral osteotomy, and the remaining cement mantle was removed with the use of a high-speed burr. There was no evidence of infection in any case. The use of an extended humeral osteotomy or fenestration of the humerus for implant removal (if well fixed) is previously described [17].

Typically, a long-stem revision reverse geometry Delta Xtend implant (DePuy, Johnson and Johnson, UK) was used to bypass the fracture and was cemented into the distal humeral fragment. The extended humeral osteotomy (if used) was closed. A cortical strut allograft (120 mm in length is generally appropriate) was used to support the implant fixation and was held in place using 5 cable ties (Dall-Miles cable system, Stryker, USA) (Fig. 3b). Fixation and implant were assessed on table for stability, and the wound closed in a standard fashion. Postoperative regime was to use an abduction wedge for 4 weeks to further prevent movement at the fracture site.

**Fig. 4:**
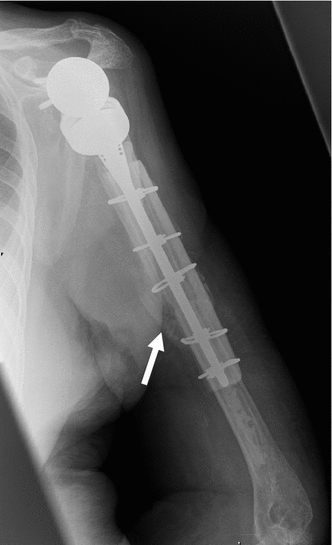
Postoperative plain radiograph of case 1 at 3 months showing well-fixed revision long-stem reverse geometry implant augmented with strut graft. Callus can clearly be seen at the fracture site (*white arrow*)

**Fig. 5:**
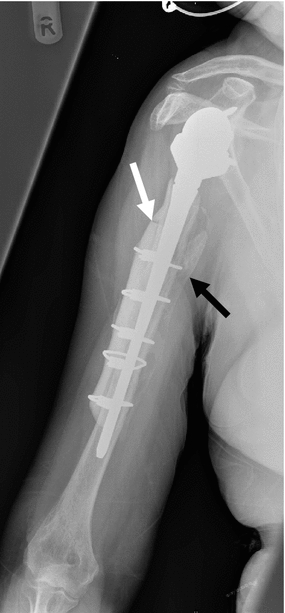
Incorporation of the biological strut allograft (*white arrow*) at 13 months postoperatively in case 2. Callus can also be seen at the fracture sites (*black arrow*)

Clinical and radiographic follow-up for all patients was carried out at 2, 8, 12–14, 20–24 weeks and approximately 1 year postoperatively. Clinical assessment included functional scoring using the Oxford shoulder score (OSS) and the disabilities of the arm, shoulder and hand score (DASH). Postoperative scores were compared to pre-operative scores. Radiographic assessment was used to confirm the presence of callus at the fracture site (Fig. 4) and incorporation of the strut allograft (Fig. 5).

Initial complications in our series of patients included one patient who developed a radial nerve neurapraxia (altered sensation and wrist drop) although this resolved entirely within 4 weeks. Later complications include a further periprosthetic fracture in one patient. This fracture was at the distal end of the construct. Although it was planned to be fixed 6 weeks later (due to the need for a CT and an available appropriate operating slot), it was found to have started to unite when screening under anaesthetic. As such, no open surgery was required.

Data are summarised in Table 1.

**Table 1:** Summary of data for several patients with revision to reverse geometry prosthesis and augmentation with cortical strut allograft for the treatment of periprosthetic humeral fractures

Case	1	2	3
Age	81	70	74
Fracture classification	Periprosthetic B2	Primary spiral fracture below malunited neck of humerus fracture	Periprosthetic B2
Treatment	Surgical	Surgical	Surgical
Primary/revision stem	Revision	Primary	Revision
Extended humeral osteotomy	Yes	No	Yes
Reverse geometry stem	Yes	Yes	Yes
Stem bypass fracture	Yes	Yes	Yes
Strut allograft	Yes	Yes	Yes
Cable ties	Yes	Yes	Yes
Time to fracture union	3 months	7 months	5 months
Time to strut incorporation	6 months	13 months	8 months
Functional result	Returned to pre-injury level	Returned to pre-injury level	Returned to pre-injury level
Complications	Transient radial nerve neurapraxia		Transient ulnar nerve neurapraxia. Further periprosthetic fracture 2/12 postop. Conservatively managed

## Discussion

Controversy still remains as to the best way to manage periprosthetic humeral fractures. Wright and Cofield [9], Campbell et al. [6] and Worland et al. [8] recommend operative management, yet Boyd et al. [18] had previously suggested conservative management although this is now largely accepted as only appropriate for type A, some B1 and certain type C fractures. Patients who have an arthroplasty in situ by definition have a compromise to shoulder function. Surely, the goal for managing patients with periprosthetic fractures must therefore revolve around restoring as much function as possible?

Boyd did show that limited glenohumeral motion led to a delay in the union of fractures at the tip of implants. He recognised that the goals (much the same as for the treatment of all fractures) in these cases were to (1) achieve union, (2) maintain glenohumeral motion and (3) restore function for the patient [18]. This is important to remember therefore in the decisions surrounding the management of patients with cuff arthropathy, a functional shoulder is required for fractures of the humeral shaft to unite. Hence our use of reverse geometry revision implants in patients with an absent or functionally deficient rotator cuff. We feel it is important therefore to take into account the patient’s pre-morbid shoulder function when planning treatment of periprosthetic fractures. If revision implants are required to help control the fracture and provide stability, then revision to reverse geometry implants in patients with cuff deficiency makes good sense.

Generally, it is accepted that periprosthetic fractures around the tips of implants are slow to unite and on the whole require operative intervention. Interestingly, as described originally by Charnley [19], fractures through the cement mantle are reported to have no adverse effect on time to union.

Complications from surgical intervention in these humeral fractures are predominantly related to radial nerve injuries (6–25 % reported) and non-union of the fracture (up to 13 %) [18]. Our results and complication rates are comparable with those described in the literature. All our complications were transient nerve injury related to mobilisation of these structures at surgery, with no deep or superficial infection reported. The patient who suffered a further periprosthetic fracture 2 months postoperatively was managed without the need for open surgery. Although the initial intention had been to fix this distal fracture, by the time the patient had their pre-operative reassessment, their CT and an appropriate slot was available in the operating theatre, 6 weeks later. The fracture was screened under image intensifier and found to be already on the way to union. Thus, the decision was made to treat without open surgery. Although the mechanical factors may have been against a fracture healing at this location, perhaps the increased biological activity at the distal end of the strut provided a suitable environment for fracture union.

Worland had average time to union of 3.3 months, in 6 patients. Forward elevation of 70° and external rotation to 38° were achieved in their study [8]. Our patient’s functional restoration was similar—all returned to their pre-morbid level. Clearly, these patients have grossly abnormal shoulder function to begin with, and the use of reverse geometry implants will not restore normal shoulder function. Reliance on deltoid muscle power to provide functional range of motion is the accepted rationale behind the use of reverse implants. Achievement of forward elevation and abduction in the region of 70–90 degrees is acceptable. Rotation will usually be limited. Our patients achieved this, and their DASH and OSS scores returned to their pre-morbid level.

Our time to fracture union ranged from 3 to 7 months. This is comparable to the cases reported in the current literature. Our times to strut graft incorporation were 6, 8 and 13 months. To date, no further complications have occurred, and at their last follow-up, all patients were doing well highlighting that the short- and medium-term results for this surgery are encouraging.

It is important to remember that cast or brace immobilisation can be used for management of postoperative fractures that occur distal to a well-fixed and stable prosthetic stem. However, cast or brace immobilisation results in fracture union but rehabilitation may be greatly impaired, and there is an increased risk of complications associated with immobilisation of the extremity [6].

As described above, the use of biological strut allograft is favourable for numerous reasons. It is questionable as to whether the strain environment provided by the use of a long revision stem bypassing the fracture site alone is adequate—even those of 220 mm. Further augmentation could be required in some cases. There is no doubt that additional cables or the use of metallic plates augmented with cables will make for a stronger construct—but this could be considered to be too stiff—the opposite end of the spectrum. However, the use of a biological material is preferred due to its Young’s modulus of elasticity being closer to that of the native bone. This reduces the risk of periprosthetic fracture at the ends of the construct by avoiding dramatic changes in stiffness along the length of the bone and thus reducing areas of stress concentration. Further advantage of cortical-only graft over metallic cable plate systems includes the relative osteoconductivity [20, 21] of the graft and the absence of metal contact on periosteum, both of which aid fracture healing. Cortical strut grafts are incorporated by creeping substitution through the process of intramembranous bone formation at the cortical junctions [21]. The immediate structural support along with the osteoconductivity of the graft makes them attractive in this scenario [22]. The incorporation of the strut grafts in our cases highlights this osteoconductive nature of our fixation.

There are of course limitations and risks to the use of biological augments in fracture fixation. The grafts themselves are expensive and are not often kept ‘on site’ in UK hospitals, as per the Human Tissue Authority Recommendations [23]. The authors have found it useful to order more than one strut graft for such cases to allow for accidental droppage/destabilisation of the graft, or fracture during trimming and sizing of the graft. The graft can also fracture during application of the cables. None of these complications occurred during the series described in this paper, but they are recognised issues with the use of cortical grafts. It is also therefore essential to ensure that a traditional cable plate system is available in reserve, should this technique be used.

Disease transmission is a major concern and risk with the use of allograft [21, 22]. Fresh allograft, typically unavailable in the UK, carries higher risk. The freezing of allograft decreases enzyme degradation and host immune response and also destroys osteogenic cells and leaving only osteoinductive capacity. Testing for HIV, Hepatitis C and often Hepatitis B is common. The risk of transmission of these diseases is low yet concerns remain over the transmission of prions. Sterilisation involves the use of gamma irradiation or ethylene oxide sterilisation. Ethylene oxide sterilisation is cheaper, but it may negatively affect the mechanical strength or biologic activity of the graft. Gamma radiation has been found to have a greater negative influence on the mechanical properties of allografts, whereas ethylene oxide affects the osteoinductive properties [21]. The freezing process for storage may also affect mechanical strength of the grafts with freeze-drying affecting the biomechanics more so than deep freezing [22].

We feel that our approach to the management of these patients focussed on restoration of shoulder function as well as fixation of the fracture. Without the use of reverse geometry implants, it is unlikely that the patients would have achieved their pre-morbid function. Although not a first-line management option, the authors can recommend consideration of this safe technique in similar situations.

As supported by the literature review, we advocate the surgical management for most types of complex humeral periprosthetic fractures. Good results can be expected if appropriate treatment is applied, paying attention to the fracture configuration and the shoulder function. Restoration of shoulder function is paramount, and therefore, the use of reverse geometry implants is recommended in those patients with rotator cuff deficiency. Failure to recognise the need for appropriate biological and mechanical environments for fracture union will lead to delay in healing and significant patient morbidity. The use of strut allograft augmentation is a new technique not yet well described in the literature. It has the potential to improve bone stock [16] and has a proven track record in the management of periprosthetic femoral fractures.

In summary, we believe revision to reverse geometry long-stem implant with or without cortical strut allograft augmentation to be safe and appropriate in the management of these complex injuries, although technically challenging, and has excellent initial to medium-term results.
